# p63 expression confers significantly better survival outcomes in high-risk diffuse large B-cell lymphoma and demonstrates p53-like and p53-independent tumor suppressor function

**DOI:** 10.18632/aging.100898

**Published:** 2016-02-14

**Authors:** Zijun Y. Xu-Monette, Shanxiang Zhang, Xin Li, Ganiraju C. Manyam, Xiao-xiao Wang, Yi Xia, Carlo Visco, Alexandar Tzankov, Li Zhang, Santiago Montes-Moreno, Karen Dybkaer, April Chiu, Attilio Orazi, Youli Zu, Govind Bhagat, Kristy L. Richards, Eric D. Hsi, William W.L. Choi, J. Han van Krieken, Jooryung Huh, Maurilio Ponzoni, Andrés J.M. Ferreri, Xiaoying Zhao, Michael B. Møller, Ben M. Parsons, Jane N. Winter, Miguel A. Piris, L. Jeffrey Medeiros, Ken H. Young

**Affiliations:** ^1^ Department of Hematopathology, The University of Texas MD Anderson Cancer Center, Houston, TX 77030, USA; ^2^ University of Indiana School of Medicine, Indianapolis, IN 46202, USA; ^3^ Department of Bioinformatics and Computational Biology, The University of Texas MD Anderson Cancer Center, Houston, TX 77030, USA; ^4^ San Bortolo Hospital, Vicenza, Italy; ^5^ University Hospital, Basel, Switzerland; ^6^ Hospital Universitario Marques de Valdecilla, Santander, Spain; ^7^ Aalborg University Hospital, Aalborg, Denmark; ^8^ Memorial Sloan-Kettering Cancer Center, New York, NY 10065, USA; ^9^ Weill Medical College of Cornell University, New York, NY 10065, USA; ^10^ The Methodist Hospital, Houston, TX 77030, USA; ^11^ Columbia University Medical Center and New York Presbyterian Hospital, New York, NY 10032, USA; ^12^ University of North Carolina School of Medicine, Chapel Hill, NC 27513, USA; ^13^ Cleveland Clinic, Cleveland, OH 44195, USA; ^14^ University of Hong Kong Li Ka Shing Faculty of Medicine, Hong Kong, China; ^15^ Radboud University Nijmegen Medical Centre, Nijmegen, Netherlands; ^16^ Asan Medical Center, Ulsan University College of Medicine, Seoul, Korea; ^17^ San Raffaele H. Scientific Institute, Milan, Italy; ^18^ Zhejiang University School of Medicine, Zhejiang, China; ^19^ Odense University Hospital, Odense, Denmark; ^20^ Gundersen Medical Foundation, La Crosse, WI 54601, USA; ^21^ Feinberg School of Medicine, Northwestern University, Chicago, IL 60611, USA; ^22^ The University of Texas School of Medicine, Graduate School of Biomedical Sciences, Houston, TX 77030, USA

**Keywords:** p63, DLBCL, p53, TP53 mutation, MDM2

## Abstract

The role of p53 family member, p63 in oncogenesis is the subject of controversy. Limited research has been done on the clinical implications of p63 expression in diffuse large B-cell lymphoma (DLBCL). In this study, we assessed p63 expression in *de novo* DLBCL samples (n=795) by immunohistochemistry with a pan-p63-monoclonal antibody and correlated it with other clinicopathologic factors and clinical outcomes. p63 expression was observed in 42.5% of DLBCL, did not correlate with p53 levels, but correlated with p21, MDM2, p16^INK4A^, Ki-67, Bcl-6, IRF4/MUM-1 and CD30 expression, *REL* gains, and *BCL6* translocation. p63 was an independent favorable prognostic factor in DLBCL, which was most significant in patients with International Prognostic Index (IPI) >2, and in activated-B-cell–like DLBCL patients with wide-type *TP53*. The prognostic impact in germinal-center-B-cell–like DLBCL was not apparent, which was likely due to the association of p63 expression with high-risk IPI, and potential presence of ∆Np63 isoform in *TP63* rearranged patients (a mere speculation). Gene expression profiling suggested that p63 has both overlapping and distinct functions compared with p53, and that p63 and mutated p53 antagonize each other. In summary, p63 has p53-like and p53-independent functions and favorable prognostic impact, however this protective effect can be abolished by *TP53* mutations.

## INTRODUCTION

*TP63*, a member of the *TP53* gene family, encodes p63 with 2 types of isoforms: a form with the N-terminal transactivation (TA) domain (TAp63) and a truncated form without the N-terminus (∆Np63). Both TAp63 and ΔNp63 have isoforms α, β, γ, δ, and ε owing to alternative splicing at the 3′ end [1–5]. p63 shares structural and sequence homology with p53 and p73, the third member of the p53 family [1, 6]. Like p53, TAp63 has been implicated in cell cycle arrest and apoptosis in response to DNA damage, ectoderm development, maternal reproduction and metabolism, dependent or independent of p53-functions [1, 7#x2013;^13^]. For example, TAp63 can transactivate some well-known p53 target genes including *CDKN1A*, *BAX* and *MDM2* [1, 14]. Moreover, p53-dependent apoptosis in response to DNA damage required p63 and p73 in mouse developing brain and embryonic fibroblasts [7]. However, in a mouse model p63 and p73 did not contribute to p53 tumor suppression function in lymphoma development [15]. ∆Np63, on the other hand, interacts with p53, TAp63, and TAp73 in a dominant-negative fashion to inhibit their tumor-suppressive functions [3]. It is generally believed that TAp63, like p53, is a tumor suppressor, whereas ∆Np63 has a critical role in epidermal development and functions as an oncogene in a mouse model [16–19]. Furthermore, the α, β, γ, δ, and ε isoforms of TAp63 and ΔNp63 have differential functions [5, 14, 20–24].

In normal human tissues, p63 expression is tissue-specific and restricted to epithelial cells, certain subpopulations of basal cells, and occasionally cells in the germinal centers of lymph nodes [1, 25, 26]. Accordingly, in tumors structural disruption of *TP63* and aberrant p63 expression are commonly seen in squamous cell and transitional cell carcinomas, but are also observed in non-Hodgkin lymphomas, predominantly in diffuse large B-cell lymphoma (DLBCL) and follicular lymphoma (grade 2 and 3) [25–30].

In basal epithelial cells and squamous cell carcinomas, the ∆Np63 isoform, especially ∆Np63α, is predominantly expressed, possibly due to the increased ∆Np63 stability caused by the lack of the transactivation domain which is indispensable for proteasome-dependent MDM2-independent degradation of p63 [24, 31]. In contrast, TAp63 is present mostly in epithelial lining cells at lower levels under normal physiological conditions, and in adenocarcinoma, thymoma and lymphoma cells; TAp63 accumulates in response to genotoxic stress [24, 26]. Although p63 expression has been shown in a few studies to indicate a poor prognosis in some carcinomas [32–34], its prognostic significance in DLBCL is unclear.

DLBCL is the most common type of non-Hodgkin lymphoma and can be divided into germinal center B-cell–like (GCB) and activated B-cell–like (ABC) subgroups by gene expression profiling [35]. Numerous genetic factors affecting the prognosis of DLBCL have been identified [36]. In our previous study, *TP53* mutations were detected in approximately 20% of *de novo* DLBCL cases and conferred a worse prognosis among DLBCL patients treated with rituximab, cyclophosphamide, doxorubicin, vincristine, and prednisolone (R-CHOP) [37]. Overexpression of mutated but not wild-type p53 (WT-p53) protein is also associated with a poor prognosis in DLBCL patients [38]. The dysregulation, expression, and clinical implications of p63 in DLBCL are less clear than those of p53; likewise, p63's role in tumorigenesis and its functional relationship with p53 are not well understood. p63, predominantly TAp63 (likely TAp63β and/or TAp63γ) but not ∆Np63 or p63α, was found expressed in 15.1% to 52.5% of DLBCLs at higher levels than in normal lymphoid tissues [21, 25–27, 39]. Truncated p63 homologous to ∆Np63 due to *TP63* gene rearrangements was also reported in 1.2%-5% of DLBCL, exclusive of GCB subtype [40, 41]. Conflicting results showing the effect of p63 expression on patients’ prognosis have been reported [21, 27, 39, 42, 43], likely owing to small number of patients (fewer than 100) in each study, the use of different cutoffs for p63 positivity, the differential functions and complicated interactions of multiple p63 isoforms [23, 43].

To fill this knowledge gap, we studied the prognostic effects of p63 expression correlating with *TP53* status in a multicenter cohort of patients with well-characterized *de novo* DLBCL treated with R-CHOP. We found that p63 expression conferred better clinical outcomes in DLBCL which however could be compromised or abolished by the difference in International Prognostic Index (IPI) scores and/or the presence of *TP53* mutations. We further investigated p63-associated biology to understand possible underlying molecular mechanisms.

## RESULTS

### p63 expression in DLBCL

**Figure 1:**
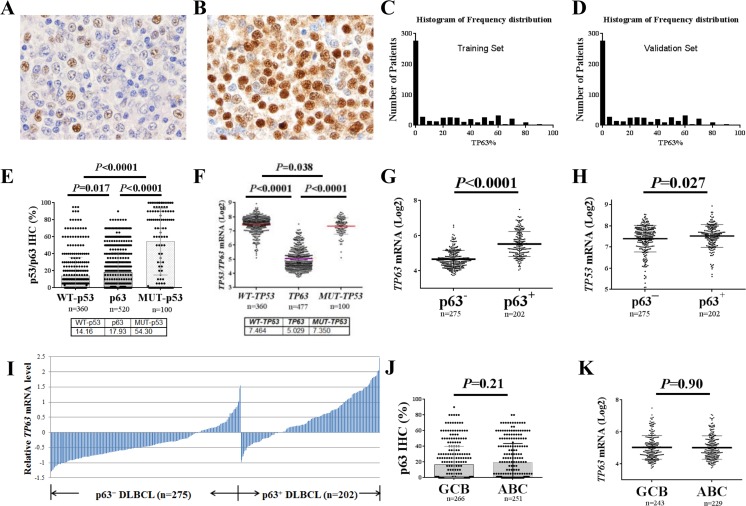
p63 expression in diffuse large B-cell lymphoma (DLBCL) in comparison with p53 expression (**A-B**) Representative immunohistochemistry staining for p63 (10% and 95%) in DLBCL. (**C-D**) Histograms of p63 expression in the training and validation sets. (**E**) Comparisons between p63 and p53 expression levels in DLBCL. (**F**) Comparisons between *TP63* and *TP53* mRNA levels in DLBCL. (**G**) p63^+^ DLBCL had significantly higher levels of *TP63* mRNA compared with p63^−^ DLBCL. (**H**) p63^+^ DLBCL had significantly higher *TP53* mRNA levels compared with p63^−^ DLBCL. (**I**) Expression of p63 protein correlated with *TP63* mRNA levels. The *TP63* mRNA expression levels (Log2 values) were retrieved from the gene expression profiling data. The mean values of 3 probe-sets (1555581_a_at, 207382_at, 209863_s_at) for each patient were used. The relative mRNA level refers to the difference between the *TP63* mRNA level for each patient and the mean *TP63* mRNA level for the entire cohort. (**J-K**) Comparisons of p63 protein and *TP63* mRNA expression levels between germinal center B-cell–like (GCB) and activated B-cell–like (ABC) subtypes of DLBCL patients.

We observed nuclear expression of p63 at variable levels in tumor cells of 317 (61%) of 520 samples from patients in the training set and 180 (65%) of 275 samples from patients in the validation set. Representative immunohistochemical stains are shown in Fig 1A, B and the histograms of p63 expression by immunohistochemistry are shown in Fig 1C, D. The mean number of p63 positive tumor cells in the training set was 18%, which was significantly higher than that of WT-p53 (*P*=0.017) but significantly lower than that of mutated p53 (MUT-p53, *P*<0.0001, Fig 1E) (Supplemental Fig 1A, B) [37, 38], although the *TP63* mRNA levels were significantly lower than the *TP53* mRNA levels (*P*<0.0001, Fig 1F). p63 protein expression significantly correlated with *TP63* mRNA (Spearman rank correlation: r=0.596, *P*<0.0001).

Owing to the significantly lower level of p63 compared with MUT-p53 expression in DLBCL and the exclusion of potential false-positive cases, we used a cutoff value of 5% of tumor cells being p63-positive for p63 expression in DLBCL (p63^+^: >5%). Using this cutoff, 221 patients (42.5%) in the training set, and 130 (47%) of 275 patients in the validation set had p63^+^ DLBCL. The p63^+^ group showed a significantly higher mean *TP63* mRNA level compared with the p63^−^ group (unpaired *t* test, *P*<0.0001, Fig 1G) and *TP53* mRNA level (Fig 1H). Transcriptional activation appeared to be the most common mechanism for p63 expression in this study of DLBCL (Fig 1I). No significant difference in the expression levels of p63/*TP63* was observed between the GCB and ABC subtypes of tumor samples, either at the protein (16.66% *vs.* 19.26%, *P*=0.21) or mRNA (*P*=0.90) levels (Fig 1J, K).

### Clinical and pathobiological features of p63^+^ DLBCL

**Figure 2:**
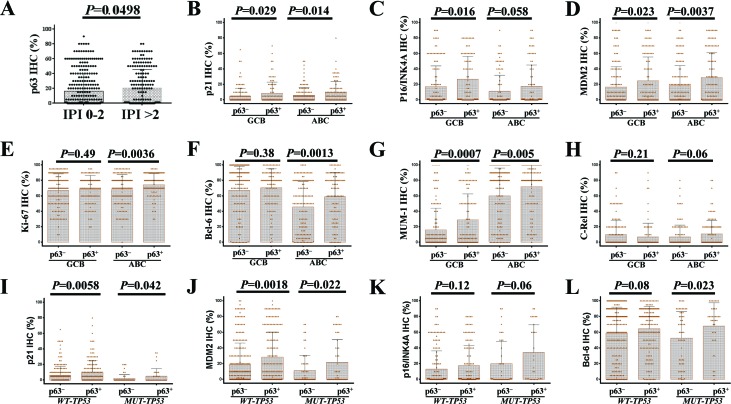
Correlations between p63 expression and other tumor associated factors (**A)** The group with high International Prognostic Index (IPI) scores had a significantly higher mean level of p63 expression. (**B-D**) p63 expression was associated with significantly higher levels of p21, MDM2, and p16-INK4a in both germinal center B-cell like (GCB) and activated B-cell like (ABC) subtypes of DLBCL patients. (**E-F**) p63 expression was associated with significantly higher levels Ki-67 and Bcl-6 in ABC-DLBCL. (**G**) p63 expression was associated with significantly higher levels of IRF4/MUM-1 in both GCB and ABC subtypes of DLBCL patients. (**H**) p63^+^ ABC-DLBCL was associated with a trend toward higher c-Rel level. (**I-J**) The association of p63 expression with p21 and MDM2 is independent of p53 mutation status. (**K-L**) p53 mutation status did not impact the association with increased p16-INK4a, Bcl-6 and IRF4/MUM-1(figure not shown) levels.

We compared the clinicopathologic features of patients with p63^+^ and p63^−^ DLBCL. The p63^+^ group more often had male (*P*=0.0056) and patients with small (< 5 cm) tumors (*P*=0.05) than did the p63^−^ group. In addition, a higher proportion (41.9%) of p63^+^ patients had an IPI score >2 compared with p63^−^ patients (34.4%), but this difference was not significant (*P*=0.086); however, by unpaired *t* test, patients with IPI scores >2 showed significantly higher mean levels of p63 (*P*=0.05, Fig 2A) and MUT-p53 (*P*=0.011, figure not shown) than did patients with IPI scores ≤ 2. When DLBCL cases were stratified into the GCB and ABC subtypes, in GCB-DLBCL p63^+^ compared with p63^−^ patients was associated with IPI scores >2, small tumors, and possibly stage III/IV disease (*P*=0.06), whereas in ABC-DLBCL p63^+^ patients had higher percentages of male gender and extranodal DLBCL (44% compared with the 31% in p63^−^ ABC-DLBCL) (Table 1). In contrast, WT-p53 overexpression was more common in nodal DLBCL (data not shown).

**Table 1:** Clinical characteristics of patients with *de novo* DLBCL in the training cohort

	DLBCL			GCB-DLBCL			ABC-DLBCL			
	p63^+^	p63^−^		p63^+^	p63^−^		p63^+^	p63^−^		
	N (%)	N (%)	*P1*	N (%)	N (%)	*P2*	N (%)	N (%)	*P3*	*P4*
**Patients**	221	299		110	156		110	141	.59	
**Age (yr)**										
< 60	92 (42)	133 (45)	.52	52 (47)	84 (54)	.29	39 (35)	47 (33)	.73	.075
≥ 60	129 (58)	166 (55)		58 (53)	72 (46)		71 (65)	94 (67)		
**Sex**										
Female	76 (34)	139 (46)	**.0056**	41 (37)	68 (44)	.30	34 (31)	70 (50)	**.0028**	.32
Male	145 (66)	160 (54)		69 (63)	88 (56)		76 (69)	71 (50)		
**Stage**										
I-II	94 (44)	141(49)	.30	50 (48)	89 (59)	**.06**	44 (41)	51 (37)	.54	.34
III-IV	119 (56)	148 (51)		55 (52)	61 (41)		63 (59)	86 (63)		
**B symptoms**										
No	136 (63)	183 (65)	.63	73 (67)	101 (70)	.67	62 (58)	81 (60)	.76	.17
Yes	79 (37)	97 (35)		35 (32)	43 (30)		44 (42)	53 (40)		
**Serum LDH**										
Normal	79 (39)	107 (39)	.97	42 (42)	56 (39)	.70	37 (36)	51 (39)	.71	.40
Elevated	124 (61)	169 (61)		58 (58)	86 (61)		65 (64)	81 (61)		
**No. of extranodal sites**										
0-1	156 (75)	228 (79)	.27	79 (78)	121 (81)	.56	76 (71)	106 (77)	.30	.23
≥ 2	53 (25)	61 (21)		22 (22)	28 (19)		31 (29)	32 (23)		
**Performance status**										
0-1	164 (85)	225 (83)	.66	78 (85)	119 (86)	.76	85 (84)	104 (79)	.35	.90
≥ 2	30 (15)	46 (17)		14 (15)	19 (14)		16 (16)	27 (21)		
**Size of largest tumor**										
< 5cm	106 (65)	120 (55)	**.05**	56 (70)	62 (55)	**.04**	49 (59)	58 (54)	.51	.14
≥ 5cm	58 (35)	99 (45)		24 (30)	50 (45)		34 (41)	49 (46)		
**IPI score**										
0-2	125 (58)	189 (66)	.086	65 (61)	111 (75)	**.025**	59 (55)	76 (56)	.90	.32
3-5	90 (42)	99 (34)		41 (39)	38 (25)		49 (45)	61 (44)		
**Therapy response**										
CR	178 (81)	227 (76)	.21	87 (79)	118 (76)	.51	90 (82)	104 (74)	.13	.61
PR	24	43		13	19		11	24		
SD	8	13		6	7		2	6		
PD	11	16		4	12		7	7		
**Primary origin**										
Nodal	131 (60)	193 (66)	.16	69 (64)	97 (64)	1.0	62 (56)	95 (69)	**.048**	.27
Extranodal	88 (40)	99 (34)		39 (36)	55 (36)		48 (44)	43 (31)		
**Ki-67**										
< 70%	66 (30)	119 (40)	**.0016**	41 (37)	64 (42)	.45	24 (22)	55 (39)	**.004**	**.018**
≥ 70%	155 (70)	175 (60)		69 (63)	88 (58)		86 (78)	86 (61)		
***TP53* mutations**										
*WT-TP53*	154 (80)	206 (77)	.65	70 (74)	105 (76)	.76	83 (85)	100 (79)	.38	.059
*MUT-TP53*	40 (21)	60 (23)		25 (26)	34 (24)		15 (15)	26 (21)		
***MYC* translocation**										
No	138 (89)	158 (88)	.86	62 (89)	73 (80)	.20	75 (89)	85 (95)	.15	1
Yes	17 (11)	22 (12)		8 (11)	18 (20)		9 (11)	4 (5)		
***BCL2* translocation**										
No	159 (84)	187 (81)	.44	68 (74)	74 (64)	.18	90 (94)	113 (97)	.31	**.0002**
Yes	30 (16)	44 (19)		24 (26)	41 (36)		6 (6)	3 (3)		
***BCL6* translocation**										
No	98 (60)	145 (74)	**.0041**	54 (69)	83 (78)	.16	43 (51)	61 (69)	**.0016**	**.016**
Yes	66 (40)	51 (26)		24 (31)	23 (22)		42 (49)	28 (31)		
***REL* gains**										
Normal	140 (86)	216 (92)	**.068**	62 (77)	118 (92)	**.003**	77 (94)	98 (92)	**.036**	**.0001**
Amplification/polysomy	23 (14)	19 (8)		18 (23)	10 (8)		5 (6)	9 (8)		
***REL* amplification**										
No	156 (95)	227 (97)	.60	72 (91)	121 (95)	.40	83 (99)	106 (99)	1.0	**.03**
Yes	8 (5)	8 (3)		7 (9)	7 (5)		1 (1)	1 (1)		
**CD30 expression**										
−	175 (79)	259 (88)	**.0048**	86 (78)	133 (88)	**.06**	88 (80)	125 (89)	**.049**	.87
+	46 (21)	34 (12)		24 (22)	19 (12)		22 (20)	15 (11)		
**p53 expression**										
< 20%	116 (61)	172 (66)	.27	57 (61)	89 (66)	.40	59 (62)	83 (66)	.48	1.0
≥ 20%	74 (39)	87 (34)		37 (39)	45 (34)		37 (38)	42 (34)		

**Abbreviations:** DLBCL, diffuse large B-cell lymphoma; GCB, germinal center B-cell–like; ABC, activated B-cell–like; LDH, lactate dehydrogenase; IPI, International Prognostic Index; CR, complete remission; PR, partial response; SD, stable disease; PD, progressive disease.

**Note:** *P* values indicate the significance of differences in the positivity frequencies of listed parameters between 2 groups. *P1* values are for comparisons between overall p63^+^ and p63^−^ DLBCL patients; *P2* values are for comparisons between p63^+^ and p63^−^ GCB-DLBCL patients; *P3* values are for comparisons between p63^+^ and p63^−^ ABC-DLBCL patients; *P4* values are for comparisons between p63^+^ GCB-DLBCL and p63^+^ ABC-DLBCL patients. For therapy response, we calculated *P* values by comparing CR to other responses.

When correlating p63 expression with other genetic abnormalities and immunohistochemical biomarkers in DLBCL, we found that the p63^+^ group had higher frequencies of *BCL6* translocation and CD30 positivity (21% compared with the 12% in p63^−^ patients) (Table 1), as well as elevated expression levels of Bcl-6, IRF4/MUM-1, p21, MDM2, p16-INK4a, and Ki-67 (in ABC-DLBCL only); most of these associations were independent of *TP53* mutation status (Fig 2B-L). In addition, p63 expression was associated with *REL* gains (including amplification and polysomies) in both the GCB and ABC subsets. No significant differences in frequencies of *TP53* mutations, *MYC* or *BCL2* translocations, or the expression levels of p53, Myc, or Bcl-2, were observed between the p63^+^ and p63^−^ groups.

### p63 expression confers better clinical outcomes, more apparently in high-risk DLBCL and ABC-DLBCL

#### Univariate survival analysis in the training set

**Figure 3:**
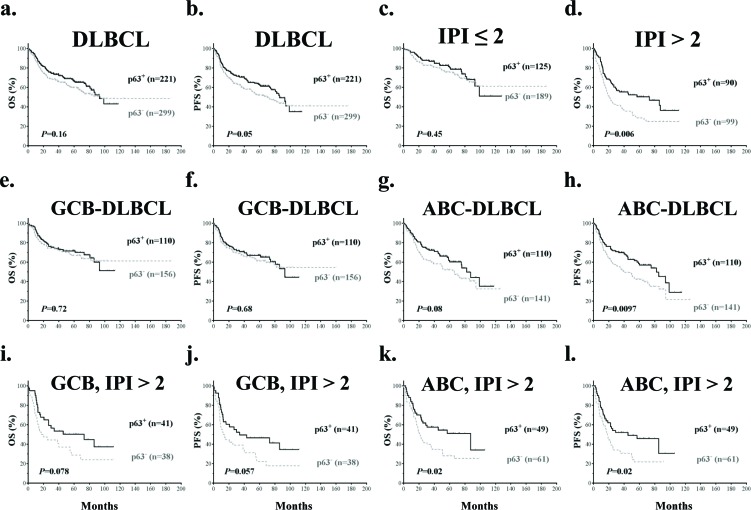
Prognostic analysis of p63 expression in DLBCL (**a-b**) p63 expression correlated with significantly better progression-free survival (PFS) but not overall survival (OS) in DLBCL. (**c-d**) p63 expression correlated with significantly better overall survival in DLBCL patients with IPI scores > 2 but not in DLBCL patients with IPI scores ≤ 2. (**e-h**) p63 expression correlated with significantly better PFS in ABC- but not GCB-DLBCL patients. (**i-j**) p63 expression was associated with trends towards better survival outcomes in GCB-DLBCL patients with IPI scores > 2. (**k-l**) p63 expression correlated with significantly better survival outcomes in ABC-DLBCL patients with IPI scores > 2.

With a median follow-up of 62 months, p63^+^ DLBCL patients showed better progression-free survival (PFS, *P*=0.05) compared with p63^−^ DLBCL patients (Fig 3a, b). When patients with low-risk (IPI score ≤ 2) and high-risk DLBCL (IPI score >2) were analyzed separately (Fig 3c, d), p63 expression showed prognostic significance only in the high-risk group and correlated with significantly better overall survival (OS) (*P*=0.006) and PFS (*P*=0.0043).

When analyzed in GCB- and ABC-DLBCL subsets separately, patients with p63^+^ ABC-DLBCL showed significantly better PFS (*P*=0.0097) and a trend of better OS (*P*=0.08) compared with p63^−^ ABC-DLBCL patients. In contrast, in GCB-DLBCL, p63 expression did not show significant impact on OS or PFS (Fig 3e-h). Consistently, survival analysis based on *TP63* mRNA levels showed that *TP63* mRNA expression correlated with favorable OS and significantly better PFS in ABC-DLBCL patients only (*P*=0.06 and *P*=0.036 respectively, Supplemental Fig S1D-G).

Since the p63^+^ GCB-DLBCL group had a higher portion of patients with high-risk DLBCL (Table 1) which may have confounded the analysis [44], patients with low-risk and high-risk DLBCL were analyzed separately. In GCB-DLBCL patients with IPI scores >2, p63^+^ GCB-DLBCL patients showed trends of better OS and PFS (*P*=0.078 and *P*=0.057 respectively) (Fig 3i-j). Similarly, the prognostic impact of p63 expression in ABC-DLBCL patients was more apparent in those with IPI scores >2 (Fig 3k-l); For ABC-DLBCL patients with IPI scores ≤ 2, OS and PFS rates were higher for p63^+^ patients but not the differences were not significant (*P*=0.48 and *P*=0.12 respectively).

#### Multivariate survival analysis

We performed multivariate survival analysis for p63 expression adjustin g clinic al parameters including IPI score, sex, tumor size and B symptoms. p63 expression was found to be an independent prognostic factor for better OS in the overall DLBCL, GCB-DLBCL and ABC-DLBCL sets, and an independent prognostic factor for better PFS in the overall DLBCL and ABC-DLBCL sets but not in the GCB-DLBCL set (Table 2).

**Table 2:** Multivariate survival analysis

	OS			PFS		
Variable	HR	95% CI	*P*	HR	95% CI	*P*
**Overall DLBCL**						
IPI >2	3.08	2.21-4.38	< **.0001**	2.84	2.08-3.89	< **.0001**
p63^+^	.62	.45-.87	.006	**.66**	.48-.90	**.009**
Female sex	.86	.62-1.20	.37	.92	.67-1.26	.60
Tumor size ≥5 cm	1.30	.94-1.79	.11	1.26	.93-1.70	.14
B symptoms present	1.32	.95-1.85	.10	1.24	.90-1.71	.18
**GCB-DLBCL**						
IPI >2	4.00	2.36-6.79	< **.0001**	3.44	2.27-5.21	< **.0001**
p63^+^	**.64**	.41-.99	.045	.67	.42-1.09	.11
Female sex	.94	.61-1.45	.78	1.00	.67-1.50	.99
Tumor size ≥5 cm	1.53	.92-2.54	.10	1.46	.92-2.34	.11
B symptoms present	1.08	.69-1.70	.74	1.21	.74-1.98	.44
**ABC-DLBCL**						
IPI >2	2.35	1.61-3.43	< **.0001**	2.23	1.57-3.16	< **.0001**
p63+	.56	.38-.83	**.004**	.58	.40-.83	**.003**
Female sex	.77	.52-1.15	.20	.78	.54-1.12	.17
Tumor size ≥5 cm	1.03	.58-1.56	.88	.99	.66-1.47	.94
B symptoms present	1.06	.72-1.58	.76	1.14	.79-1.64	.49
**DLBCL with *WT-TP53***						
IPI >2	3.29	2.21-4.88	< **.0001**	3.21	2.18-4.72	< **.0001**
p63^+^	.61	.40-.91	**.015**	.63	.43-.92	**.016**
p53^+^	.97	.62-1.52	.90	.91	.60-1.40	.68
Female sex	.91	.61-1.36	.65	.85	.57-1.26	.42
Tumor size ≥5 cm	1.19	.81-176	.38	1.11	.76-1.62	.59
B symptoms present	1.45	.97-2.17	.07	1.48	1.00-2.20	**.049**
**DLBCL with *MUT-TP53***						
IPI >2	2.43	1.17-5.05	**.017**	2.11	1.07-4.18	**.032**
p63^+^	.70	.34-1.44	.33	.72	.36-1.44	.36
p53^+^	3.16	1.17-8.52	**.023**	2.30	.97-5.45	.06
Female sex	1.02	.50-2.11	.96	1.12	.57-2.20	.75
Tumor size ≥5 cm	1.57	.77-3.20	.21	1.85	.95-3.63	.07
B symptoms present	1.19	.54-2.60	.67	1.03	.49-2.17	.93

Abbreviations: DLBCL, diffuse large B-cell lymphoma; ABC, activated B-cell–like; OS, overall survival; PFS, progression-free survival; HR, hazard ratio; CI, confidence interval; IPI, International Prognostic Index.

#### Validation set

Similar to the training set, no significant difference was observed in p63 expression between the GCB and ABC subtypes (*P*=0.68). These similar prognostic impacts as in the training set were all significant with a ≥5% cutoff value for p63 expression (*P*=0.02, *P*=0.047, and *P*=0.0007 for PFS in DLBCL, ABC-DLBCL and high-risk DLBCL respectively. Supplemental Fig S2). A multivariate survival analysis indicated that after adjusting clinical parameters, p63 expresssion ≥5% was an independent favorable prognostic factor in overall DLBCL and ABC-DLBCL but not in GCB-DLBCL (data not shown).

### Relationships with *TP53* mutations and p53 expression

#### Non-significant correlation with p53 expression and correlation with TP53 mRNA

**Figure 4:**
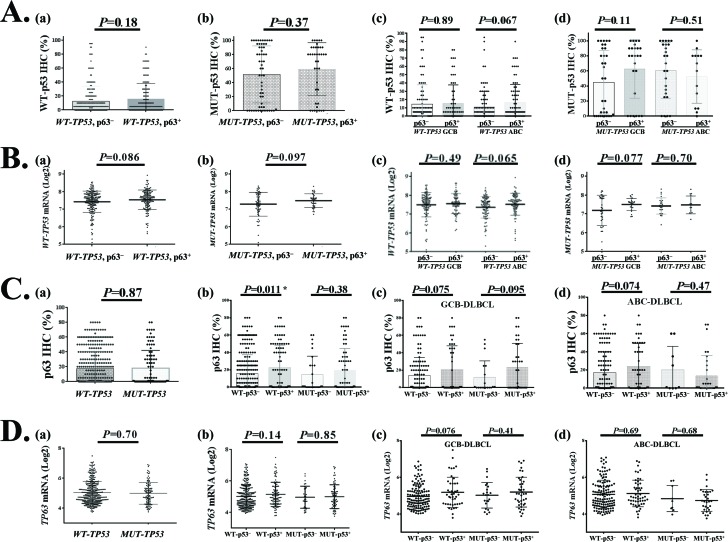
Relationship between *TP63*/p63 and *TP53*/p53 expression in DLBCL (**A**) Comparison of wild-type (WT) or mutated (MUT) p53 protein expression between p63^+^ and p63^−^ patients with DLBCL or GCB/ABC subtypes of DLBCL. (**B**) Comparison of *TP53* mRNA expression between p63^+^ and p63^−^ patients with *WT-TP53* or *MUT-TP53* and GCB/ABC DLBCL. (**C**) Comparison of p63 protein expression between *WT-TP53* and *MUT-TP53* DLBCL, and between p53^+^ and p53^−^ patients with DLBCL or GCB/ABC subtypes of DLBCL. (**D**) Comparison of *TP63* mRNA expression between *WT-TP53* and *MUT-TP53* DLBCL, and between p53^+^ and p53^−^ patients with DLBCL or GCB/ABC subtypes of DLBCL.

By Spearman rank correlation *TP63* mRNA showed correlation with *TP53* mRNA levels in the overall DLBCL set (r=0.091, *P*=0.048) and *WT-TP53* subset (r=0.106, *P*=0.044) but not in the *MUT-TP53* subset. In contrast, p63 expression did not show significant correlation with overall p53 (r=0.071, *P*=0.132), WT-p53 (r=0.08, *P*=0.135), or MUT-p53 (r=0.072, *P*=0.481). Using unpaired *t*-tests, p63 expression did not correlate with p53 levels (Supplemental Fig S1C), but was associated with elevated *TP53* mRNA levels (Fig 1H). Analysis in GCB/ABC DLBCL subsets with WT-p53 or MUT-p53 showed no significant correlations between p63 positivity and WT-p53/MUT-p53 expression levels (Table 1, Fig 4A, B). However, the WT-p53^+^ (≥20% [38]) compared with the WT-p53^−^ DLBCL group had a significantly higher mean level of p63 protein (Fig 4C(b)) but not *TP63* mRNA (Fig 4D).

#### Prognostic impact of p63 expression in the presence of WT-TP53 or MUT-TP53

**Figure 5:**
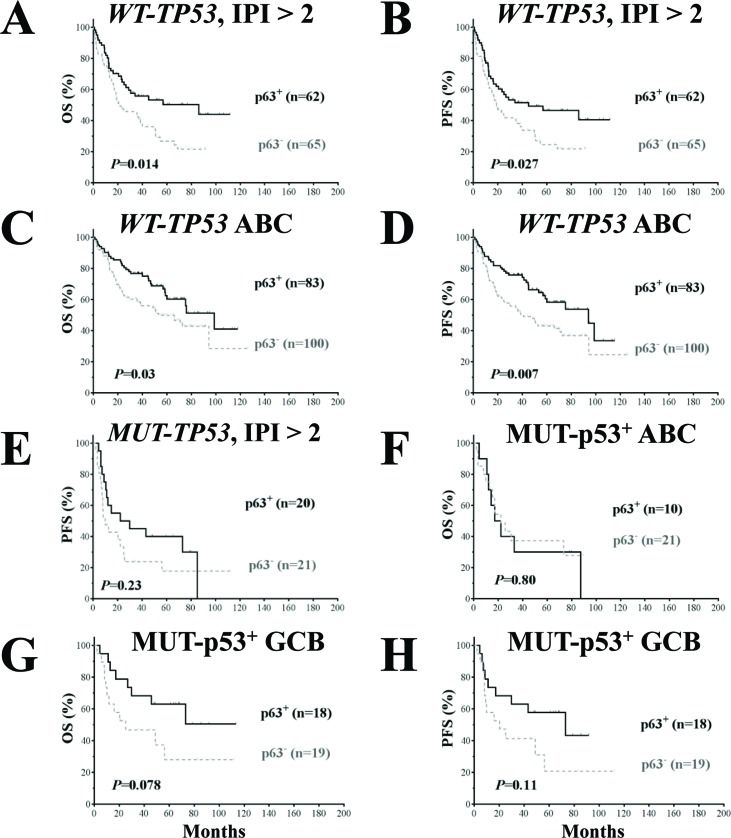
Prognostic analysis for p63 expression in DLBCL patients with wild- type and mutated *TP53* (**A-B**) p63 expression correlated with significantly better survival outcomes in patients with high-risk (IPI > 2) DLBCL and *WT-TP53*. (**C-D**) p63 expression correlated with significantly better survival outcomes in patients with ABC-DLBCL and *WT-TP53*. (**E**) In patients with high-risk (IPI > 2) DLBCL and *MUT-TP53*, p63 expression did not correlate with survival outcomes although showed a trend toward better PFS. (**F**) p63 expression did not correlate with survival outcomes in patients with ABC-DLBCL and MUT-p53 overexpression. (**G-H**) p63 expression was associated with trends toward better survival outcomes in GCB-DLBCL patients with MUT-p53 overexpression (marginal *P* values).

The clinicopathologic features of patients with p63^+^ or p63^−^ DLBCL with *WT-TP53* or *MUT-TP53* are shown in Table 3. p63 expression was associated with significantly better OS and PFS in patients with *WT-TP53* and IPI scores >2 (Fig 5A, B) and in ABC-DLBCL patients with *WT-TP53* (Fig 5C, D), and favorable trends in patients with MUT-p53^+^ GCB-DLBCL (Fig 5G, H).

**Table 3:** Clinical characteristics of DLBCL patients with wild-type *TP53* (*WT-TP53*) or mutated *TP53* (*MUT-TP53*)

	*WT-TP53*			*MUT-TP53*		
	p63^+^	p63^−^		p63^+^	p63^−^	
Characteristic	N (%)	N (%)	*P*	N (%)	N (%)	*P*
**Patients**	154	206		40	60	
**Age (y)**						
< 60	62 (40)	87 (42)	.75	16 (40)	25 (42)	1.0
≥ 60	92 (60)	119 (58)		24 (60)	35 (58)	
**Gender**						
Male	106 (69)	107 (52)	**.0013**	24 (60)	36 (60)	1.0
Female	48 (31)	99 (48)		16 (40)	24 (40)	
**Stage**						
I-II	62 (42)	97 (49)	.21	17 (43)	28 (47)	.68
III-IV	84 (58)	100 (51)		23 (58)	32 (53)	
**B symptoms**						
No	100 (67)	196 (66)	.92	23 (59)	38 (68)	.37
Yes	50 (33)	65 (34)		16 (41)	18 (32)	
**LDH**						
Normal	58 (41)	82 (44)	.66	12 (32)	19 (33)	.93
Elevated	82 (59)	105 (56)		25 (68)	38 (67)	
**No. of extranodal sites**						
0-1	105 (73)	155 (78)	.30	29 (74)	46 (78)	.68
≥ 2	38 (27)	43 (22)		10 (26)	13 (22)	
**Performance status**						
0-1	117 (87)	231 (85)	.62	30 (88)	50 (86)	.78
≥ 2	18 (13)	28 (15)		4 (12)	8 (14)	
**Size of largest tumor**						
< 5cm	81 (68)	90 (58)	.079	18 (58)	23 (48)	.38
≥ 5cm	38 (32)	66 (42)		13 (42)	25 (52)	
**IPI risk group**						
0-2	87 (58)	132 (67)	.10	19 (49)	38 (64)	.12
3-5	62 (42)	65 (33)		20 (51)	21 (36)	
**Therapy response**						
CR	126 (82)	163 (79)	.59	27 (68)	35 (58)	.40
PR	16	24		7	13	
SD	3	7		3	3	
PD	9	12		3	9	
**Ki-67**						
< 70%	50 (33)	83 (41)	.10	9 (22)	17 (29)	.64
≥ 70%	104 (67)	119 (59)		31 (78)	42 (71)	
**Primary origin**						
Nodal	91 (40)	134 (66)	.21	25 (37)	39 (70)	.54
Extranodal	61 (60)	68 (66)		15 (63)	17 (30)	
**DLBCL subtypes**						
GCB	70 (46)	105 (51)	.34	25 (62)	34 (57)	.68
ABC	83 (54)	100 (49)		15 (38)	26 (43)	
***BCL6* translocation**						
–	73 (63)	97 (70)	.21	17(57)	35 (85)	**.007**
+	43 (37)	41 (30)		13(43)	6 (15)	
**CD30**						
−	120 (78)	178 (89)	**.0085**	32 (80)	54 (92)	.13
+	34 (22)	23 (11)		8 (20)	5 (8.5)	
**p53 expression**						
< 20%	106 (70)	154 (77)	.18	10 (26)	18 (31)	.65
≥ 20%	45 (30)	47 (23)		29 (74)	40 (69)	

**Abbreviations**: DLBCL, diffuse large B-cell lymphoma; R-CHOP, rituximab with cyclophosphamide, doxorubicin, vincristine, and prednisone; LDH, lactate dehydrogenase; IPI, international prognostic index; CR, complete remission; PR, partial response; SD, stable disease; PD, progressive disease; GCB, germinal center B-cell–like; ABC, activated B-cell–like.

#### Multivariate survival analysis

We further performed multivariate survival analysis including p63 expression, p53 overexpression, and clinical parameters in the *WT-TP53* and *MUT-TP53* subsets individually. In the *WT-TP53* subset, p63 expression but not WT-p53 overexpression remained as an independent prognostic factor for better OS and PFS; in the *MUT-TP53* subset, MUT-p53 overexpression but not p63 expression was an independent prognostic factor for poorer PFS (borderline *P* value for OS) (Table 2).

### Gene expression profiling signature of p63 expression

**Figure 6:**
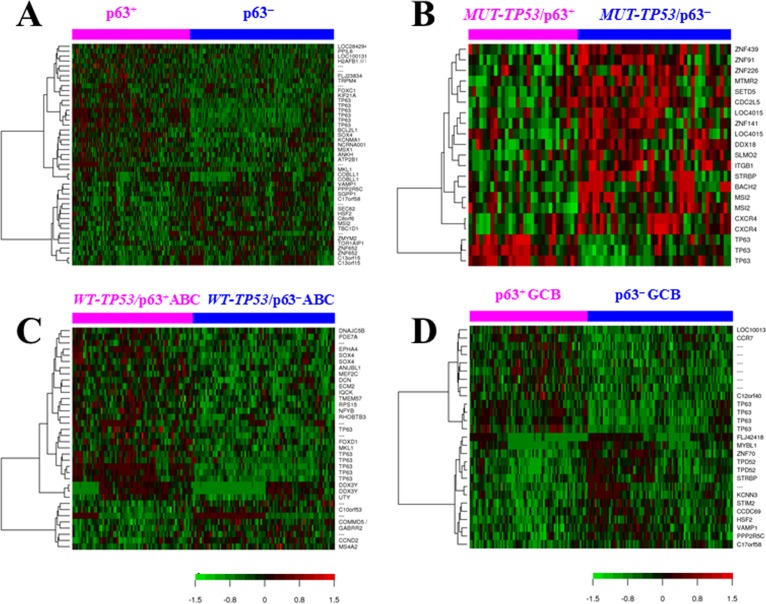

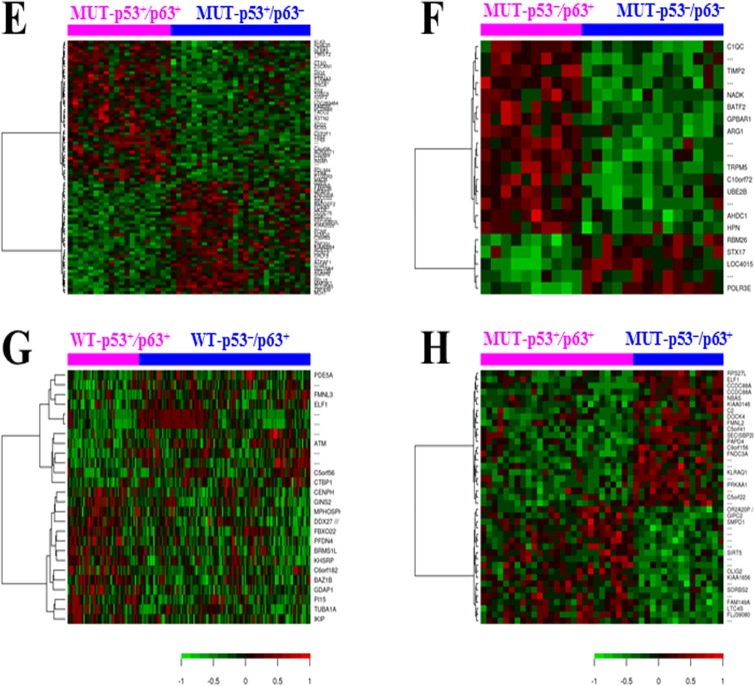
Gene expression profiling analysis (**A**) Heatmap for comparison between p63^+^ and p63^−^ DLBCL patients (false discovery rate < 0.15). (**B**) Heatmap for comparison between p63^+^ and p63^−^ DLBCL patients with *MUT-TP53* (false discovery rate < 0.05, fold change >1.68). (**C**) Heatmap for comparison between p63^+^ *versus* p63^−^ patients with ABC-DLBCL and *WT-TP53* (false discovery rate < 0.20). (**D**) Heatmap for comparison between p63^+^ *versus* p63^−^ patients with GCB-DLBCL (false discovery rate < 0.05). (**E**) Heatmap for comparison between p63^+^ and p63^−^ DLBCL patients with MUT-p53 overexpression (false discovery rate < 0.15). (**F**) Heatmap for comparison between p63^+^ and p63^−^ DLBCL patients with no or low (< 20%) expression levels of MUT-p53 (false discovery rate < 0.10). (**G**) Heatmap for comparison between WT-p53^+^ (≥ 20%) and WT-p53^−^ (< 20%) DLBCL patients with p63 expression (false discovery rate < 0.30). (**H**) Heatmap for comparison between MUT-p53^+^ (≥ 20%) and MUT-p53^−^ (< 20%) DLBCL patients with p63 expression (false discovery rate < 0.20).

To gain insights into the potential molecular mechanisms underlying the prognostic observation, we performed a series of GEP analyses comparing p63^+^ and p63^−^ patients in the overall DLBCL group and various subsets stratified by GCB/ABC subtype, *TP53* mutation and p53 overexpression status (Fig 6A-H, Supplemental Fig S3A-D). Counts of significant differentially expressed genes (DEGs) between compared groups with different false discovery rate (FDR) thresholds are listed in Supplemental Table S1. Largely, whether p63 expression was associated with distinct GEP signatures did not correlated with whether p63 showed apparent prognostic effects, and the GEP signature of p63 expression in the *MUT-TP53* subset was much more prominent (Fig 6B, Table 4) than that in the *WT-TP53* subset (7 genes only with a FDR threshold of 0.30, figure not shown). However, after dividing the *WT*-*TP*53 subset into GCB and ABC subtypes of DLBCL patients, p63 expression showed GEP signatures, more distinctive in ABC than in GCB (Fig 6C, Supplemental Fig S3A), which was opposite to the pattern for overall ABC and GCB (only few DEGs in ABC compared to the distinct GEP signature in GCB, Supplemental Table S1). The p63 GEP signatures in the *MUT-TP53* and *WT-TP53* subsets had both similarity (upregulated *ATP2A2* and downregulated *ZNF652*) and difference (three genes, *GABBR2*, *PDHA1* and *NFYB*, showed opposite up- or down-regulation). Reinforcing the idea that p63 GEP signatures are more highlighted in the absence of WT-p53 activities as shown in the *MUT-TP53* subset, we further found that in WT-p53^−^ ABC-DLBCL but not in WT-p53^+^ ABC-DLBCL, p63 expression was associated with significant DEGs (Supplemental Fig S3B, Supplemental Table S3).

**Table 4:** Genes differentially expressed between patients with p63^+^ and p63^−^ DLBCL

	p63^+^ DLBCL FDR< 0.20		p63^+^ DLBCL with *MUT*-*TP53* FDR< 0.05, fold change > 1.68		p63^+^ ABC-DLBCL with *WT*-*TP53* FDR< 0.20	
Function categories	Upregulated	Downregulated	Upregulated	Downregulated	Upregulated	Downregulated
Signaling, immune response, inflammation	***FLJ23834, TRAF1***	*SGPP1*			***FOXD1, PDE7A***	*GABRR2, MS4A2, COMMD5*
Development, differentiation	***SOX4, FOXC1***			*ZNF141, BACH2*	***SOX4, EPHA4***	
Cell growth and proliferation, gene expression, metabolism	***H2AFB1/2/3***	*MSI2, TBC1D1, ZNF652, TOR1AIP1, ZMYM2*		*STRBP, CDC2L5, DDX18, MSI2, ZNF439, ZNF91, ZNF226, MTMR2*	***MEF2C, DCN, KDM2B, RPS15, NFYB, DDX3Y, FOXD1*,*****UTY***	*CCND2*
Apoptosis, cell death, DNA damage response	***TP63, BCL2L1, ZAK, RFFL, ATG4B, MKL1, HIPK2***	*C13orf15*	***TP63***		***TP63, MKL1***	
Protein folding, protein translocation, heat shock	***PPIL6***	*HSF2, SEC62*				
Transport, mobility, cell adhesion	***KCNMA1, ATP2B1, KIF21A, ANKH, TRPM4***	*VAMP1*		*ITGB1, CXCR4*	***ECM2, RHOBTB3***	
IncRNA and other unknown function	***COBLL1, NCRNA00173***	*C17orf58, C8orf6*		*SETD5, SLMO2*	***DNAJC5B, TMEM57, ANUBL1, IQCK***	*C10orf53*

**Abbreviations**: DLBCL, diffuse large B-cell lymphoma; FDR, false discovery rate; IncRNA, long noncoding RNA.

To gain insights into the functional relationship between p53 and p63, we further analyzed the overlap and difference between the p53 [37, 38] and p63 GEP signatures. The results (Table 5) suggest p63 expression had a WT-p53-like GEP signature either in the context of *WT-TP53* (such as *CTAG2*, *SOX4* and *ELL2*, accounting for approximately 21% of the DEGs between *WT-TP53*/p63^+^ and *WT-TP53*/p63^−^) or *MUT-TP53* (such as *DSE*, *ATM*, *CDK13*, *CD47*, *ELF1*, *DYRK1A* [45], *PFDN4*, and *TMEM97*, accounting for approximately 4% of the DEGs between *MUT-TP53*/p63^+^ and *MUT-TP53*/p63^−^), yet remained some MUT-p53-like GEP signature mainly in the context of *MUT-TP53* (such as *CAMTA1* resembling the MUT-p53 GEP signature, and *ABHD11*, *KCNN3*, *MART3*, and *MRPL30* opposite to the WT-p53 GEP signature; accounting for approximately 1.4% of the DEGs between *MUT-TP53*/p63^+^ and *MUT-TP53*/p63^−^). Moreover, only in the p63^+^ but not in the p63^−^ subset, expression of WT-p53 or MUT-p53 was associated with distinct GEP signatures (Fig 6G, H), which may suggest that p63 is important for p53 activities.

**Table 5:** Lists of differentially expressed genes between p63^+^ and p63^−^ DLBCL that are also in the p53 signatures and MDM2 signatures

Common genes shared by the p63^+^ and p53^+^ signatures				
	***WT-TP53* WT-p53^+^*vs* WT-p53**^−^		***MUT-TP53* MUT-p53^+^*vs* WT-p53^−^*MUT-TP53 vs WT-TP53***	
		Same		Same
**Up** ↑	***DSE***	↑ in *MUT-TP53/*p63^+^ *vs MUT-TP53/*p63^−^ ↑ in MUT-p53^+^*/*p63^+^ *vs* MUT-p53^+^*/*p63^−^	***BCAS1***	↑in p63^+^ GCB *vs* p63^−^ GCB
	***ELL2***	↑ in *WT-TP53/*p63^+^ *vs WT-TP53/*p63^−^		
	***FDXR***	↑ in WT-p53^−^/p63^+^ ABC *vs* WT-p53^−^/p63^−^ ABC		
	***GRRP1***	↑ in *MUT-TP53/*p63^+^ *vs MUT-TP53/*p63^−^		
	***HPGD***	↑ in *MUT-TP53/*p63^+^ *vs MUT-TP53/*p63^−^		
	***PFDN4***	↑ in *MUT-TP53/*p63^+^ *vs MUT-TP53/*p63^−^		
	***SOX4***	↑ in p63+ *vs* p63^−^ ↑ in *WT-TP53/*p63^+^ *vs WT-TP53/*p63^−^ ↑in WT-p53^−^/p63^+^ ABC *vs* WT-p53^−^/p63^−^ ABC		
**Down** *↓*	***ATM***	*↓* in *MUT-TP53/*p63^+^ *vs MUT-TP53/*p63^−^	***CAMTA1***	*↓* in *MUT-TP53/*p63^+^ *vs MUT-TP53/*p63^−^ *↓* in *MUT-TP53/*p63^+^ GCB *vs MUT-TP53/*p63^−^ GCB
	***C3orf63***	*↓* in MUT-p53^+^*/*p63^+^ *vs* MUT-p53^+^*/*p63^−^		
	***CCDC69***	*↓* in p63^+^ GCB *vs* p63^−^ GCB		
	***CD47***	*↓* in MUT-p53^+^*/*p63^+^ *vs* MUT-p53^+^*/*p63^−^		
	***CDC2L5/CDK13***	*↓* in *MUT-TP53/*p63^+^ *vs MUT-TP53/*p63^−^ *↓* in *MUT-TP53/*p63^+^ GCB *vs MUT-TP53/*p63^−^ GCB		
	***DCLRE1C***	*↓* in *MUT-TP53/*p63^+^ *vs MUT-TP53/*p63^−^ *↓* in *MUT-TP53/*p63^+^ GCB *vs MUT-TP53/*p63^−^ GCB		
	***DYRK1A***	*↓* in *MUT-TP53/*p63^+^ *vs MUT-TP53/*p63^−^		
	***ELF1***	*↓* in *MUT-TP53/*p63^+^ *vs MUT-TP53/*p63^−^ *↓* in *MUT-TP53/*p63^+^ GCB *vs MUT-TP53/*p63^−^ GCB *↓* in p63^+^ GCB *vs* p63^−^ GCB		
	***ESR2***	*↓* in p63^+^ GCB *vs* p63^−^ GCB		
	***HCG18***	*↓* in *WT-TP53/*p63^+^ *vs WT-TP53/*p63^−^		
	***HERC4***	*↓* in *MUT-TP53/*p63^+^ *vs MUT-TP53/*p63^−^ *↓* in *MUT-TP53/*p63^+^ GCB *vs MUT-TP53/*p63^−^ GCB		
	***ITCH***	*↓* in *MUT-TP53/*p63^+^ *vs MUT-TP53/*p63^−^		
	***LOC645513***	*↓* in *MUT-TP53/*p63^+^ *vs MUT-TP53/*p63^−^		
	***ORC4L***	*↓* in *MUT-TP53/*p63^+^ *vs MUT-TP53/*p63^−^		
	***PPP1R2***	*↓* in *MUT-TP53/*p63^+^ *vs MUT-TP53/*p63^−^		
	***TBC1D1***	*↓* in p63+ *vs* p63^−^*↓* in p63^+^ GCB *vs* p63^−^ GCB		
	***PXK***	*↓* in p63^+^ GCB *vs* p63^−^ GCB		
	***TMCC1***	*↓* in *MUT-TP53/*p63^+^ ABC *vs MUT-TP53/*p63^−^ ABC		
	**ZCCHC7**	*↓* in *MUT-TP53/*p63^+^ *vs MUT-TP53/*p63^−^		
	***ZNF221***	*↓* in WT-p53^−^/p63^+^ ABC *vs* WT-p53^−^/p63^−^ ABC		
		Opposite		Opposite
**Up ↑**	***KCNN3***	*↓* in p63^+^ GCB *vs* p63^−^ GCB	***CTAG2***	*↓* in WT-p53^−^/p63^+^ ABC *vs* WT-p53^−^/p63^−^ ABC
	***KIAA0564***	*↓* in *MUT-TP53/*p63^+^ *vs MUT-TP53/*p63^−^ *↓* in MUT-p53^+^*/*p63^+^ *vs* MUT-p53^+^*/*p63^−^	***TMEM97***	*↓* in *MUT-TP53/*p63^+^ *vs MUT-TP53/*p63^−^
	***MATR3***	*↓* in *MUT-TP53/*p63^+^ *vs MUT-TP53/*p63^−^	***SLC16A1***	*↓* in p63^+^ GCB *vs* p63^−^ GCB
	***MRPL30***	*↓* in *MUT-TP53/*p63^+^ *vs MUT-TP53/*p63^−^		
**Down** *↓*	***ABHD11***	↑in *MUT-TP53*/p63^+^ ABC *vs MUT-TP53*/p63^−^ ABC		
Common genes shared by the p63^+^ and MDM2^+^ signatures				
	***WT-TP53* MDM2^+^*vs* MDM2^−^**		***MUT-TP53* MDM2^+^*vs* MDM2^−^**	
		Same		Same
**Up** ↑	***FAM83A***	↑ in *MUT-TP53/*p63^+^ ABC *vs MUT-TP53/*p63^−^ ABC		
	***FDXR***	↑in WT-p53^−^/p63^+^ ABC *vs* WT-p53^−^/p63^−^ ABC		
	***MICAL2***	↑in *MUT-TP53/*p63^+^ GCB *vs MUT-TP53/*p63^−^ GCB		
	***PCBP3***	↑in *MUT-TP53/*p63^+^ *vs MUT-TP53/*p63^−^		
	***TCEB3***	↑Rin p63+ *vs* p63^−^		
**Down** *↓*	***ATM***	*↓* in *MUT-TP53/*p63^+^ *vs MUT-TP53/*p63^−^		
	***BPTF***	*↓* in *MUT-TP53/*p63^+^ *vs MUT-TP53/*p63^−^ *↓* in p63^+^ GCB *vs* p63^−^ GCB	***ATG7***	*↓* in p63^+^ GCB *vs* p63^−^ GCB
	***BRWD1***	*↓* in p63^+^ GCB *vs* p63^−^ GCB	***ATP5C1***	*↓* in *MUT-TP53/*p63^+^ *vs MUT-TP53/*p63^−^ *↓* in *MUT-TP53/*p63^+^ GCB *vs MUT-TP53/*p63^−^ GCB
	***CD22***	*↓* in p63^+^ GCB *vs* p63^−^ GCB	***EIF2A***	*↓* in *MUT-TP53/*p63^+^ *vs MUT-TP53/*p63^−^
	***DHX36***	*↓* in *MUT-TP53/*p63^+^ *vs MUT-TP53/*p63^−^	***PAK2***	*↓* in *MUT-TP53/*p63^+^ *vs MUT-TP53/*p63^−^
	***EIF2A***	*↓* in *MUT-TP53/*p63^+^ *vs MUT-TP53/*p63^−^	***PRICKLE4/TOMM6***	*↓* in *MUT-TP53/*p63^+^ ABC *vs MUT-TP53/*p63^−^ ABC
	***NKTR***	*↓* in *MUT-TP53/*p63^+^ *vs MUT-TP53/*p63^−^ *↓* in MUT-p53^+^*/*p63^+^ *vs* MUT-p53^+^*/*p63^−^ *↓* in *MUT-TP53/*p63^+^ GCB *vs MUT-TP53/*p63^−^ GCB		
	***RBM26***	*↓* in *MUT-TP53/*p63^+^ *vs MUT-TP53/*p63^−^ *↓* in MUT-p53^−^*/*p63^+^ *vs* MUT-p53^−^*/*p63^−^		
	***RPL34***	*↓* in *MUT-TP53/*p63^+^ *vs MUT-TP53/*p63^−^		
	***SLC35F5***	*↓* in *MUT-TP53/*p63^+^ *vs MUT-TP53/*p63^−^		
	***WT-TP53* MDM2^+^*vs MUT-TP53* MDM2**^+^			
		Same		
**Down** *↓*	***LPP***	*↓* in p63^+^ GCB *vs* p63^−^ GCB		

We also compared the p63 GEP signature with the MDM2 GEP signature [38], and found 21 DEGs were common between the GEP signatures of p63 and MDM2 expression, among which 16 DEGs were not shared by the p53 GEP signature (Table 5).

Although the p53 and p63 GEP signatures overlapped, majority of the DEGs were not shared. Nonetheless, a p53-like tumor suppressor role of p63 was suggested by the p63 GEP signatures, including downregulation of *CCND2* (in *WT-TP53*/p63^+^ ABC-DLBCL), *CDC27* and *MYCT1* (in *WT-TP53*/p63^+^ GCB-DLBCL), *CDC2L5/CDK13* and *CXCR4* (in *MUT-TP53*/p63^+^ DLBCL. *TP53* mutations were associated with increased CXCR4 levels especially in GCB- DLBCL as previously reported [46]), *ELF1* which encodes a transcription factor that activates *LYN* and *BLK* (in *MUT-TP53/*p63^+^ GCB-DLBCL), *MYBL1* and *STRBP* which play roles in proliferation and growth (in *MUT-TP53/*p63^+^ GCB-DLBCL), antiapoptotic *C9orf82* and *BCOR* (which encodes an interacting corepressor of BCL6 required for germinal center formation and may influence apoptosis) (in *MUT-TP53/*p63^+^ ABC-DLBCL), as well as upregulation of *HIPK2* (which promotes apoptosis through the activation of p53/*TP53*) (in p63^+^ DLBCL) and *WWOX* (which functions synergistically with p53/*TP53* to control genotoxic stress-induced cell death) (in *MUT-TP53/*p63^+^ ABC-DLBCL) (Supplemental Fig S3C-D).

On the other hand, some DEGs promoting tumor cell survival were also shown in the comparison between overall p63^+^ and p63^−^ DLBCL patients, which may be due to the oncogenic function provided by MUT-p53 or p63 isoforms in the p63^+^ DLBCL subsets. For example, antiapoptotic *BCL2L1*, *RFFL* (which negatively regulates p53, CASP8 and CASP10 through proteasomal degradation), *ATG4B* (required for autophagy), and *MKL1* (which suppresses TNF-induced cell death by inhibiting caspase activation) were up-regulated in p63^+^ DLBCL compared with p63^−^ DLBCL, whereas *C13orf15/RGCC* (in response to DNA damage) was downregulated in p63^+^ DLBCL patients (Table 4). Cytokine/receptor genes *IL17RC*, *IL4*, *IL4I1* and *IL8RB/CXCR2* which have been associated with poorer prognosis in cancers, were upregulated in *MUT-TP53/*p63^+^ compared with *MUT-TP53/*p63^−^ DLBCL (Supplemental Table S2); *MLL2* was upregulated in p63^+^ patients with ABC-DLBCL and *MUT-TP53* (Supplemental Fig S3D).

## DISCUSSION

Abnormal p63 expression patterns instead of *TP63* mutations have been found to be important for tumorigenesis [5]. Little data are available with conflicting results regarding p63 expression and its prognostic role [27, 39, 43]. We found that p63 expression correlated with a superior survival in ABC-DLBCL with *WT-TP53* and in high-risk (IPI >2) DLBCL (regardless GCB or ABC), which is consistent with a previous study in high-intermediate and high risk DLBCL [27]. The association of p63 expression with high-risk IPI in GCB-DLBCL, and thus affecting its apparent prognostic effects in GCB and overall DLBCL, may contribute to the inconsistent findings from previous studies.

The prognostic effect of p63 expression suggests that p63 has a tumor suppressor role for DLBCL, although its protective effect can be antagonized or abolished by *TP53* mutations and high-risk DLBCL associated biology. In our cohort, p63 expression was associated with increased levels of IRF4/MUM-1, p21, MDM2, and p16-INK4a resembling that of WT-p53 yet independent of p53 mutation status. GEP analysis showed that compared to the prominent p63 GEP signature within the *MUT-TP53* subset, the comparison between p63^+^ and p63^−^ patients with *WT-TP53* had much fewer DEGs; DEGs were shown within the WT-p53^−^ but not WT-p53^+^ ABC-DLBCL subset. These results may suggest that the tumor suppressor function of p63 may overlap with (and is probably weaker than) that of WT-p53, and when *TP53* was mutated, p63 functions as a supplemental tumor suppressor alternative to WT-p53. However, MUT-p53 function remained or dominated p63 function in certain *MUT-TP53* cases (Table 5), likely due to the significantly higher levels of MUT-p53 than p63 [47]. In addition to the GEP results as above, p63 expression correlated with *MDM2* upregulation and *BCL2* and *MDM4* downregulation (*P*=0.0174, *P*=0.0487 and *P*=0.090 respectively) resembling WT-p53 expression GEP signature (although the FDRs for the comparison between p63^+^ and p63^−^ DLBCL were higher). In contrast, *CDKN1A/p21*, *MCL1*, *B2M*, and *FYB* showed great variation even opposite up/down regulation between the WT-p53^+^ and the p63^+^ GEP signature. These phenomena may be explained by the remained MUT-p53-like function in the *MUT-TP53*/p63^+^ cases, whereas *TP63* mutations and expression of different p63 isoforms may not be significant factors as suggested by the previous studies [5, 25, 43] and our preliminary data of *TP63* mutations in DLBCL (unpublished data).

These observations in DLBCL may support previous functional studies, which showed that TAp63α and TAp63γ (but not ΔNp63) could induce apoptosis at lesser levels than WT-p53 [48]; TAp63, and also TAp73, together with p53, may transactivate a group of common target genes in response to DNA damage, including damage resulting from exposure to doxorubicin, a component of R-CHOP;^1^ TAp63 and MUT-p53 antagonize each other mainly in the regulation of metastasis and tumor dissemination [5]; p53 mutants may bind directly to p63 and inhibit the p63-mediated transcription of p53 target genes [49, 50]. Strategies to overcome MUT-p53 interaction with p63, decrease MUT-p53 levels and enhance p63 levels may have therapeutic value [47]. On the other hand, in mouse embryonic fibroblasts, p63 and p73 are required for p53-dependent apoptosis in response to DNA damage [7]. This may explain why our GEP comparisons between p53^+^ and p53^−^ DLBCL showed DEGs within the p63^+^ but not p63^−^ subset. Moreover, our data suggested that p63 act together with p53 in some essential pathways yet also function independently in many processes such as development, immune response and chemokinesis. Large variations between p63 signatures in the overall DLBCL patient population and in the GCB and ABC subsets may also imply a wide range of p63 activities. These characteristics of p63 function compared with p53, as well as association with high Ki-67 (consistent with previous studies [21, 43]) and high IPI may explain the limitation of p63's apparent prognostic effect in DLBCL.

It is also possible that the correlation between p63 expression and better survival outcomes may be also influenced by the escape from MDM2-mediated degradation. In our cohort, the p63's protective effects on patient survival were independent of MDM2 expression, yet GEP signatures were only shown in MDM2^low^ but not in MDM2^high^ subsets (data not shown), suggesting that MDM2 may suppress p63 function but the suppression is not significant to the p63's protective effect. Conversely, p63 may have confounded the MDM2's prognostic effect in DLBCL just as that of WT-p53 [38], suggested by the common genes shared by the MDM2 and p63 GEP signatures (Table 5). Previous studies have suggested that p63 degradation is independent of MDM2 [24, 31] and that MDM2 increases the protein level and transcriptional activity of p63 [51]. The MDM2 inhibitor p14ARF directly interacts with and impairs p63 transcriptional activity [52]. On the other hand, it has also been shown that MDM2 transports p63 out of nucleus and inhibits its transcription function [53].

**Figure 7:**
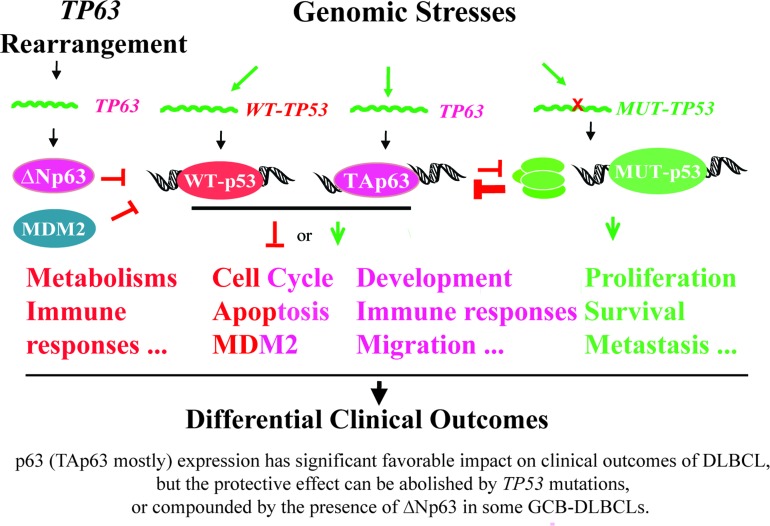
A hypothetical model illustrating the regulation and roles of p53 and p63 in DLBCL lymphomagenesis and clinical outcomes suggested by our clinical and biological data

Yang *et al*. speculated that p63 expression in cancer cells was due to *TP63* gene amplification by genomic instability [3], and other researches showed that p63 expression was regulated via mRNA stability [4, 19]. *TP63* rearrangements have been reported in 1.2-5% of DLBCL (exclusive of GCB subtype) and also in 5.8% of peripheral T-cell lymphomas, which resulted in a truncated p63 protein lacking the TA domain [40, 41]. Our data showed the associations of p63 expression with *BCL6* (mapped to 3q27) translocations, which appears to suggest the possibility of concurrent translocation of *TP63* gene (mapped to 3q27-28) due to chromosomal proximity in p63^+^ DLBCL subsets. In these cases it is possible that expressed p63 had oncogenic function like ∆Np63, which may explain the oncogenic DEGs in the p63 GEP signatures, and the lack of p63's prognostic significance in GCB-DLBCL. In addition, genomic stress similar to that inducing p53 may also be the cause of p63 expression in subsets of p63^+^ DLBCL [54], since our data showed correlation between the *WT*-*TP53* and *TP63* mRNA levels, and both WT-p53 and p63 expression were associated with increased IRF4/MUM-1 and Ki-67 expression. Fig 7 illustrates these potential causes for p63 expression and possible relationships between p63 and WT-p53/MUT-p53 function. Understanding the mechanisms regulating *TP63* may lead to therapeutic strategies. In DLBCL cell lines, FOXP1, directly represses *TP63* and cooperate with NF-κB signaling to promote lymphoma cell survival [42]. Consistently, our GEP data also suggest that molecules related to B-cell receptor signaling may be potential targets which suppresses p63 expression, as in GCB-DLBCL and *MUT-TP53/*p63^+^ DLBCL, p63 expression was associated with downregulation of *SYK* and *ELF1* respectively (suggesting decreased B-cell receptor signaling).

In conclusion, we demonstrated the correlation of p63 expression and better survival outcomes in patients with high-risk DLBCL, ABC-DLBCL with *WT-TP53*, and biology associated with p63 expression supporting p63's tumor suppressor role in DLBCL. This study helps identify a subgroup of patients with better prognosis among patients who have ABC-DLBCL or high-risk DLBCL. Targeting p63 expression and function may be a novel therapeutic strategy for particular subgroups of DLBCL patients.

## MATERIALS AND METHODS

### Patients

A total of 795 patients with *de novo* DLBCL from 20 medical centers treated with R-CHOP were studied, randomly divided into a training set (n=520) and a validation set (n=275). The diagnostic criteria, selection process, therapy, and treatment response have been described previously [37]. The study was approved as being of minimal or no risk or as exempt by the institution review boards of all participating medical centers.

### Immunohistochemistry

Tissue microarrays prepared from the diagnostic formalin-fixed, paraffin-embedded (FFPE) tissue blocks of all patients studied were stained with an anti-p63 antibody (4A4, Santa Cruz Biotechnology, Santa Cruz, CA) which can detect all p63 isoforms. Expression levels of p63 were determined by estimating the percentage of p63-positive tumor cells in the tissue array cores. X-tile software and receiver operating characteristic curve analysis by GraphPad Prism 6 Software were used to determine the percentage of p63-positive cells with maximal discriminatory power for the separation of DLBCL patients into 2 different prognostic groups. Evaluation of other biomarkers by immunohistochemistry was also performed on tissue microarrays using corresponding antibodies: p53 (DO-7, Dako, Carpinteria, CA), MDM2 (IF2, Calbiochem, Billerica, MA), p21 (Dako), Bcl-2 (Clone-124, Dako, Carpinteria, CA), Ki-67 (Dako), CD30 (clone BerH2, Dako), Bcl-6 (Dako), FOXP1 (Abcam), IRF4/MUM1 (Dako), CD10 (56C6, Vantana), c-Rel (Dako), and CXCR4 (Abcam, San Francisco, CA). Details of immunohistochemistry procedures and scoring processes have been described previously [38, 44, 55–58].

### *TP53* and *TP63* sequencing, fluorescence *in situ* hybridization

Genomic DNA samples were extracted from FFPE tissues, and the *TP53* coding region and splice site sequence were determined for 460 patients in the training set using a p53 AmpliChip (Roche Molecular Systems, Pleasanton, CA) as described previously [37]. *TP63* coding region sequence was analyzed by Sanger sequencing method. *MYC*, *BCL2*, *BCL6*, and *REL* gene arrangements and copy number aberrations were detected by fluorescence *in situ* hybridization [56, 59, 60].

### Gene expression profiling

Gene expression profiling was performed on Affymetrix GeneChips HG-U133 Plus Version 2.0 (Affymetrix, Santa Clara, CA) using total RNAs as described previously [37, 55]. The CEL files are deposited in the National Center for Biotechnology Information Gene Expression Omnibus repository (GSE#31312). The microarray data were quantified and normalized by the frozen robust multiarray analysis (RMA) algorithm. The differentially expressed genes were identified by using multiple *t*-tests.

### Statistical analysis

The clinical and pathologic features at the time of presentation were compared between various DLBCL subgroups by using the chi-square test and unpaired *t* test. Correlation between expression of different genes or proteins was evaluated by Spearman rank correlation. Overall survival (OS) was calculated from the date of diagnosis to the date of last follow-up or death. Progression-free survival (PFS) was calculated from the date of diagnosis to the date of disease progression or death. OS and PFS curves of the various groups were analyzed by GraphPad Prism 6 software using the Kaplan-Meier method, and differences were compared with use of the log-rank (Cox-Mantel) test. Multivariate analysis was conducted by using the Cox proportional hazards regression model with the SPSS software version 19.0 (IBM, Armonk, NY). Any difference with a *P* value of < 0.05 was considered statistically significant.

## SUPPLEMENTAL TABLES AND FIGURES


