# Supplementary material for: p63 expression confers significantly better survival outcomes in high-risk diffuse large B-cell lymphoma and demonstrates p53-like and p53-independent tumor suppressor function
Source: Aging (Albany NY). 2016 Feb 14;8(2):345–65. doi: 10.18632/aging.100898 (PMC4789587; doi:10.18632/aging.100898)

SUPPLEMENTAL TABLES AND FIGURES

Supplemental Table S1. Summary of gene expression profiling analysis results listing counts of significant differentially expressed transcripts between defined two groups

(A) DLBCL

FDR threshold	DLBCL p63$^+$ *vs* p63$^-$		*WT-TP53* DLBCL p63$^+$ *vs* p63$^-$		*MUT-TP53* DLBCL p63$^+$ *vs* p63$^-$	
	***P* cutoff**	**Count**	***P* cutoff**	**Count**	***P* cutoff**	**Count**
0.01	0.000001	6	0.000001	6	0.000011	11
0.05	0.000018	16	0.000009	7	0.000910	441
0.10	0.000072	26	0.000020	8	0.006131	1947
0.15	0.000168	45	0.000033	11	0.018714	4280
0.20	0.000316	63	0.000049	13	0.041307	7335
0.25	0.000534	89	0.000068	15	0.076337	11025
0.30	0.000842	135	0.000091	17	0.126082	15192

(B) GCB-DLBCL

FDR threshold	GCB-DLBCL p63$^+$ *vs* p63$^-$		*WT-TP53* GCB-DLBCL p63$^+$ *vs* p63$^-$		*MUT-TP53* GCB-DLBCL p63$^+$ *vs* p63$^-$	
	***P* cutoff**	**Count**	***P* cutoff**	**Count**	***P* cutoff**	**Count**
0.01	0.000001	5	0.000000	0	0.000000	0
0.05	0.000026	27	0.000000	0	0.000000	0
0.10	0.000251	122	0.000000	3	0.000013	8
0.15	0.000971	293	0.000002	4	0.000114	21
0.20	0.002615	571	0.000008	6	0.000525	83
0.25	0.005777	995	0.000025	8	0.001723	264
0.30	0.011279	1640	0.000065	13	0.004555	651

(C) ABC-DLBCL

FDR threshold	*WT-TP53* ABC-DLBCL p63$^+$ *vs* p63$^-$		WT-p53$^-$ ABC-DLBCL p63$^+$ *vs* p63$^-$		*MUT-TP53* ABC-DLBCL p63$^+$ *vs* p63$^-$	
	***P* cutoff**	**Count**	***P* cutoff**	**Count**	***P* cutoff**	**Count**
0.01	0.000000	3	0.000000	3	0.000000	0
0.05	0.000000	6	0.000000	4	0.000003	0
0.10	0.000004	9	0.000006	8	0.000071	8
0.15	0.000035	14	0.000058	15	0.000465	62
0.20	0.000163	36	0.000301	54	0.001767	266
0.25	0.000547	95	0.001079	162	0.004974	666

0.30	0.001496	223	0.003063	393	0.011587	1430

(D) *WT-TP53*

**FDR threshold**	In WT-p53$^+$ *vs* WT-p53$^-$ subsets, p63$^+$ *vs* p63$^-$		In MUT-p53$^-$ subset, p63$^+$ *vs* p63$^-$		In MUT-p53$^+$ subset, p63$^+$ *vs* p63$^-$	
	***P* cutoff**	**Count**	***P* cutoff**	**Count**	***P* cutoff**	**Count**
0.01	0.000000	0	0.000000	0	0.000000	0
0.05	0.000000	0	0.000001	0	0.000025	7
0.10	0.000000	0	0.000019	4	0.000377	83
0.15	0.000000	0	0.000156	21	0.001852	337
0.20	0.000000	0	0.000697	109	0.005728	915
0.25	0.000000	0	0.002229	319	0.013750	1981
0.30	0.000000	0	0.005758	758	0.028125	3620

(E) *WT-TP53*

**FDR threshold**	WT-p53$^+$ *vs* WT-p53$^-$		In p63$^-$ subset, WT-p53$^+$ *vs* WT-p53$^-$		In p63$^+$ subset, WT-p53$^+$ *vs* WT-p53$^-$	
	***P* cutoff**	**Count**	***P* cutoff**	**Count**	***P* cutoff**	**Count**
0.01	0.000000	0	0.000000	0	0.000000	0
0.05	0.000045	35	0.000000	0	0.000000	0
0.10	0.000389	175	0.000000	0	0.000000	0
0.15	0.001431	407	0.000000	0	0.000000	0
0.20	0.003705	823	0.000000	0	0.000019	1
0.25	0.007930	1379	0.000000	0	0.000069	11
0.30	0.015063	2233	0.000000	0	0.000204	26

(F) *MUT-TP53*

**FDR threshold**	MUT-p53$^+$ *vs* WT-p53$^-$		In p63$^-$ subset, MUT-p53$^+$ *vs* MUT-p53$^-$		In p63$^+$ subset, MUT-p53$^+$ *vs* MUT-p53$^-$	
	***P* cutoff**	**Count**	***P* cutoff**	**Count**	***P* cutoff**	**Count**
0.01	0.000001	6	0.000000	0	0.000000	0
0.05	0.000105	88	0.000000	0	0.000000	0
0.10	0.000730	319	0.000000	0	0.000008	0
0.15	0.002350	679	0.000000	0	0.000079	14
0.20	0.005545	1209	0.000000	0	0.000392	41
0.25	0.011049	1929	0.000000	0	0.001356	140
0.30	0.019812	2884	0.000000	0	0.003736	410

Abbreviations: DLBCL, diffuse large B-cell lymphoma; FDR, false discovery rate; GCB, germinal center B-cell like; ABC, activated B-cell like; WT, wild-type; MUT, mutated.

Supplemental Table S2. Significant differentially expressed genes between p63$^+$ and p63$^-$ DLBCL with *MUT-TP53* (false discover rate < 0.05)

	# of probes	# of genes	p63$^+$ *vs* p63$^-$ *MUT-TP53* DLBCL<br>FDR < 0.05
**Upregulated**	238	184	*TP63, PDLIM4, ASMTL, ARSD, MAGED4/MAGED4B, IL4I1, CALR, MYH6/MYH7, TCF15, LOC729234, PREX1, TSPAN4, BAIAP2, MTHFR, PTP4A3, SNCA, GRASP, CMKLR1, ARHGEF2, DOK1, SDC4, COL7A1, PRG2, KCNQ2, TRPC4AP, IL8RB, PPP2CB, FAM26F, CDK6, FBLL1, RAI1, KIAA0427, ITPRIPL2, SOBP, PFDN4, SNX11, IL17RC, GRRP1, FBF1, CBS, RARS2, VPS53, CCL27, ATP2A2, ZMYND10, CCDC149, SLC5A8, ISLR2, OSBPL5, RGN, PARP10, BST1, C14orf56, B3GNT8, DKFZP547L112, PSMD4, FPGS, TFRC, SPDEF, KIAA1652, USP36, SEMA6B, CADM4, C21orf2, CTSD, C1orf183, SSTR3, COL27A1, DIO2, SFTPC, PEAR1, LOC100293983/MUC5AC, TEAD2, AMOT, NOS3, ABHD12, C22orf31, LYPD1, KCP, PPARGC1A, MGC16025, SDK2, AHDC1, PCSK1N, MED16, RNF24, FOXN4, RIN1, BIRC7, LOC390205, LOC389043, PARD3B, NHSL1, PAX2, CYTH3, TWIST2, C8orf58/PDLIM2, KLK12, ELK4, AKT1S1, OR7A17, CEMP1, GGT7, MSLN, LOC145757, GABBR2, TMEM132A, RCN3, TRPV4, S1PR2, RHOV, GPATCH3, CTNS, SPEF1, POU5F1B, TYMP, ADAMTSL1, PCBP3, ANGPTL4, C16orf35, C14orf180, LOC728288, PRRT2, FLT4, TLE6, PRKCA, ARMC8, C16orf42, EPN1, UBTD1, MAP1LC3A, SLC39A14, TUSC5, CHRD, KIF19, C1orf46, C7orf69, WFDC2, TM6SF2, LOC91450, C14orf176, HPGD, C9orf7, LOC100128893, KIF25, DIO3OS, SLC31A1, WDR72, FLJ36208, DPYSL4, KCTD15, TREML4, LOC100292376, CTBP2, ENO3, METTL12, LTBP4, KLK3, NYX, PCDHB6, PCDHA6, NEU4, FBXO31, LOC404266, MARK2, NUDT2, C10orf72, THBD, GLP1R, UCHL5, LOC338667, ZFP36L1, ZPLD1, ADRA1D, IL4, LOC401134, BPESC1, CYP4Z1, PTGER3, CIDEA, PREX2, LIPC*
**Downregulated**	203	175	*SETD5, STRBP, ITGB1, ZNF141, ZNF91, MSI2, BACH2, SLMO2, DDX18, CDC2L5, LOC401504, ZNF439, CXCR4, MTMR2, ZNF226, LOC100130458, CFDP1, VPS35, RNF138, SLC25A36, BPTF, ZDHHC20, COX11, PM20D2, IPO5, TMEM97, RBM15, RERE, LYRM7, RBM27, ATM, LOC220930, ATP5C1, BDP1, PIGP, RC3H2, FNTA, LOC100292841/SUZ12/SUZ12P, ARHGAP15, SNHG6,*

	PCNP, RCBTB1, ALS2CR8, EIF2A, NMD3, RBM26, KBTBD2, TAF9B, LUC7L3, SMC5, SMAD2, CEBPG, TTC3, ZCCHC7, SENP5, CCDC75, PSIP1, PRPF4B, PAK2, PPP1R2, FAM13B, PPP2R5C, ZHX2, IAH1, ZNF638, NKTR, CLPX, ALG8, C19orf2, LOC645513, POLR2K, ARL5B, MRPL30, LRCH3, PRKRA, SET, SLC16A4, TPR, TOPBP1, UBR5, HAT1, ZNF460, TARDBP, MAP3K7, LOC641298, ITCH, PRPF39, SNX30, DDX17, PHF10, ELF1, UPF2, YY1, CCDC7, CTDSPL2, C1orf124, CRLF3, DYNC1LI1, MRPS30, LRRC58, FCHSD2, SPEN, KPNA1, SPOPL, ADNP, GKAP1, UNK, SLC35F5, LOC254128, CAMK2D, CCAR1, TTC37, PUM1, ZBTB44, CPSF6, NFYB, ZNF33B, MLLT10, TOP2B, DPY30/MEMO1, GPD1L, ANKHD1/ANKHD1-EIF4EBP3, YWHAB, EBAG9, RAD21, RPL22, TRAPPC2 TRAPPC2P1, TSN, MATR3, AMY2B/RNPC3, STX17, PDCD6IP, PPPDE1, KAT2B, KIAA0564, BTF3L4, LMBRD1, GLUD2, ACTR8, TTC14, RRAGC, NCBP1, ATG3, SSB, FXR1, YWHAZ, FLJ10213, CNBP, PDS5B, RPL23A, SETD2, HERC4, KIAA2026, PAPOLA, CCNL1, MLH1, DCLRE1C, MCCC1, RPL34, C1orf27, ZNF709, VPRBP, ZSCAN29, C17orf80, LOC100129064/LOC100131360/LOC100131868, PPP4R2, GTF2F2, VPS13B, PIGL, TMEM216, LOC400713, CCNL1, AKIRIN1, ZNF253, TAF7, ORC4L

Note: The order of gene lists is based on the fold changes.

Supplemental Table S3. Significant differentially expressed genes between p63^{+} and p63^{-} patients with WT-p53^{-} ABC-DLBCL (false discovery rate < 0.20)

Function categories	Upregulated	Downregulated
Signaling, immune response, inflammation	***FOXD1***	*GPR182*, *SOX14*, *TPO*, *CTAG2*, *ADORA1*, *RNASE7*, *OR5AK4P*
Development, differentiation	***SOX4***	
Cell growth and proliferation, gene expression, metabolism	***KLHDC5*, *NONO*, *PCNT*, *KDM2B*, *RRM1*, *PRPS2*, *PDHA1*, *FDXR*, *UTY*, *RBM10***	*CCND2*, *ZNF221*, *ZNF287*
Apoptosis, cell death, DNA damage response	***TP63*, *MKL1***	
Protein folding, protein translocation, heat shock	***CCT3*, *AGBL5***	
Transport, mobility, cell adhesion	***WDR66*, *TIMM9*, *NLGN4Y***	*ATP11A*
IncRNA and other unknown function	***DNAJC5B*, *CCDC141*, *TMEM106C*, *WDR41***	*C6orf59*, *NEAT1*, *CLDND1*, *QRICH2*

SUPPLEMENTAL FIGURE LEGENDS

Supplemental Figure S1. (**A-B**) In patients with *WT-TP53*, the mean expression level of p63 was significantly higher than that of WT-p53; in contrast, in patients with *MUT-TP53*, the mean expression level of p63 was significantly lower than that of MUT-p53. (**C**) The $p63^+$ DLBCL group showed trends of higher p53 protein compared with the $p63^-$ DLBCL group, but the *P* values was not significant. (**D-G**) Prognostic analysis for *TP63* mRNA expression levels in DLBCL patients. Patients were initially divided into 3 groups for survival analysis according to the mean values of *TP63* mRNA expression: low *TP63* mRNA (< mean – standard deviation), high *TP63* mRNA (> mean + standard deviation), and intermediate *TP63* mRNA levels (the remaining cases). Since there were no significant difference in survival outcomes between the low *TP63* mRNA and intermediate *TP63* mRNA groups, these two groups were combined together to compare with the high *TP63* mRNA group. (**D-E**) *TP63* mRNA levels did not correlate with survival in GCB-DLBCL significantly. (**F-G**) High *TP63* mRNA levels correlated with significantly better progression-free survival (PFS) and a strong trend of better overall survival (OS) in ABC-DLBCL patients.

Supplemental Figure S2. Prognostic analysis for p63 expression in the validation DLBCL cohort. (**A**) With a cutoff value of ≥5% for $p63^+$, p63 expression was associated with significantly better PFS in overall DLBCL set and subsets with ABC-DLBCL or with International Prognostic Index (IPI) scores >2. (**B**) With a cutoff value of >5% for $p63^+$, p63 expression was associated with significantly better PFS in patients with ABC-DLBCL, and a strong trend toward better PFS in patients with IPI scores >2.

Supplemental Figure S3. Gene expression profiling analysis of p63 expression correlating with *TP53* status. (**A**) Genes significantly differentially expressed between $p63^{+}$ and $p63^{-}$ patients with GCB-DLBCL and *WT-TP53* (false discovery rate < 0.30). (**B**) Genes significantly differentially expressed between $p63^{+}$ and $p63^{-}$ ABC-DLBCL patients with no or low (< 20%) levels of wild-type p53 expression (false discovery rate < 0.20). (**C**) Genes significantly differentially expressed between $p63^{+}$ and $p63^{-}$ patients with GCB-DLBCL and *MUT-TP53* (false discovery rate < 0.20). (**D**) Genes significantly differentially expressed between $p63^{+}$ and $p63^{-}$ patients with ABC-DLBCL and *MUT-TP53* (false discovery rate < 0.15)

Supplemental Fig. S1

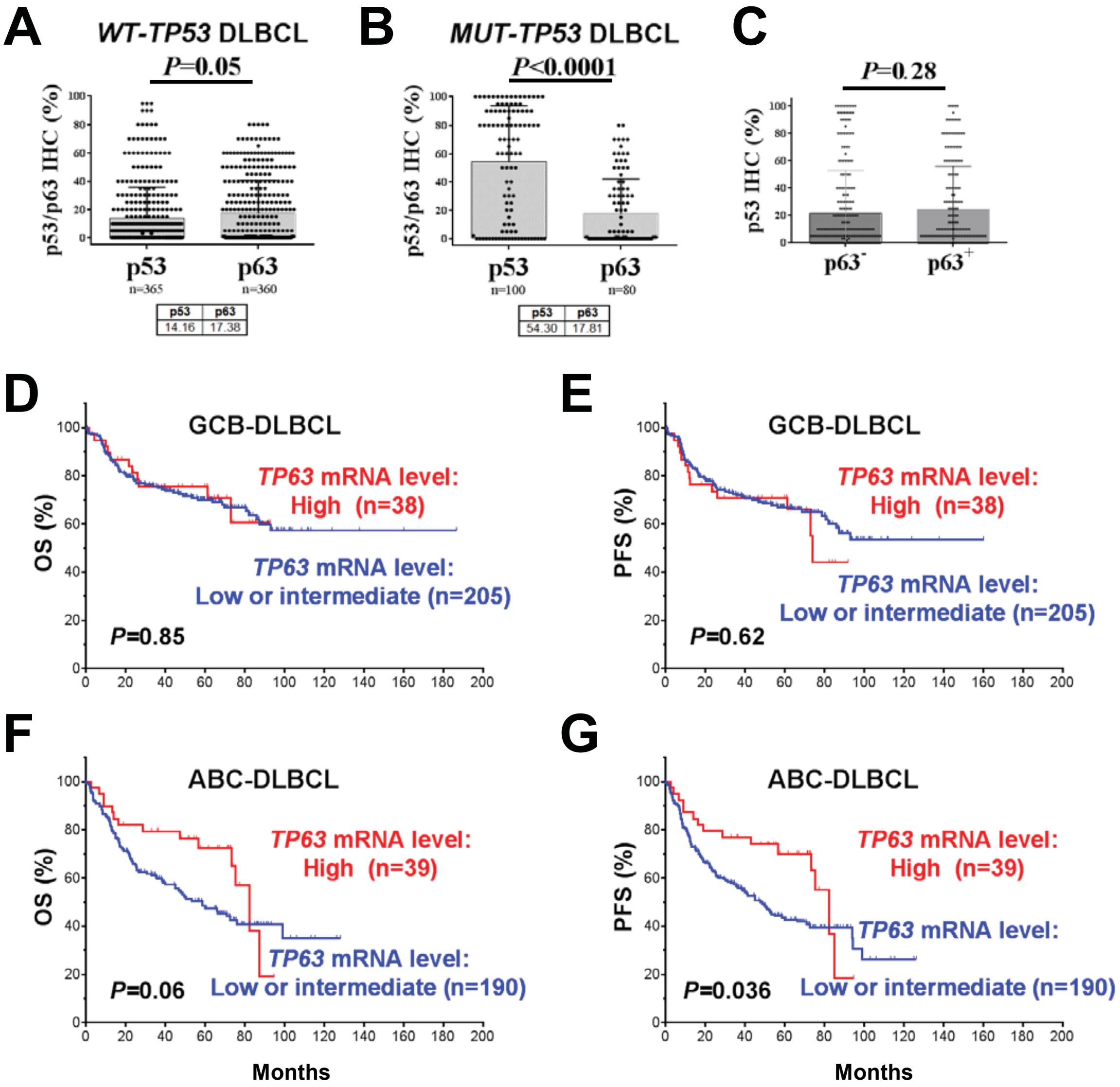

Supplemental Fig. S2

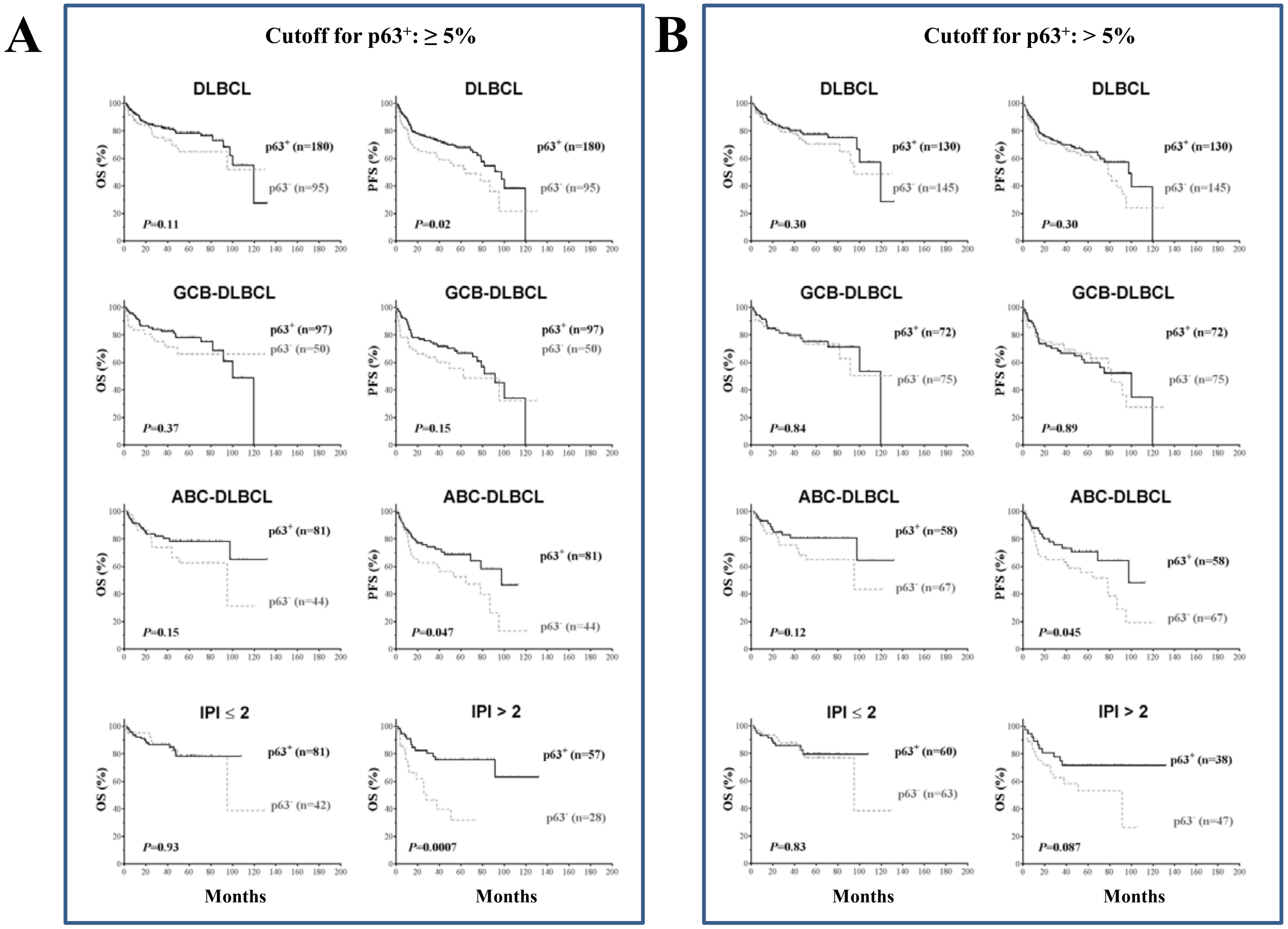

Supplemental Fig. S3

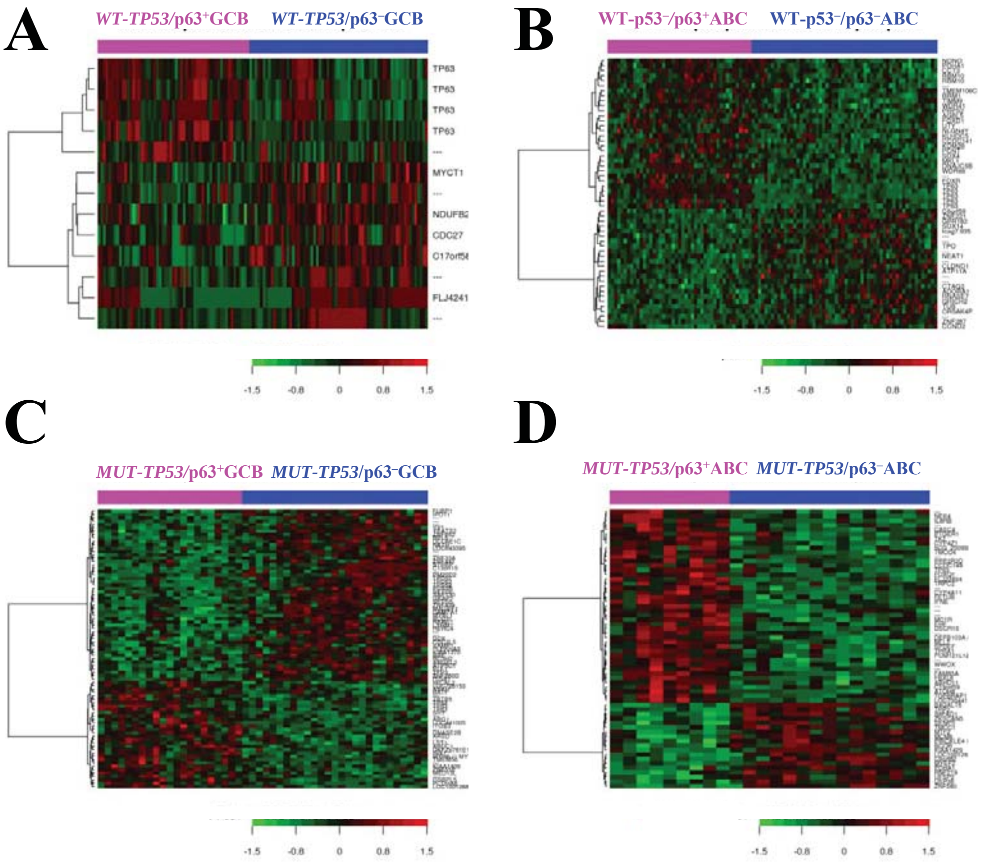

Supplement: Supplementary file 1 [file aging-08-345-s001.pdf]
